# Children’s perception of interpersonal coordination during joint painting

**DOI:** 10.1038/s41598-022-22516-2

**Published:** 2022-11-07

**Authors:** Rotem Abraham, Noemí Grinspun, Tal-Chen Rabinowitch

**Affiliations:** 1 The School of Creative Arts Therapies, University of Haifa, 3498838 Haifa, Israel; 2 Metropolitan University of Educational Sciences, 7760197 Santiago, Chile

**Keywords:** Psychology, Human behaviour

## Abstract

Interpersonal coordination is important for many joint activities. A special case of interpersonal coordination is synchronization, which is required for the performance of many activities, but is also associated with diverse positive social and emotional attributes. The extent to which these effects are due to the reliance on synchrony for task performance or to its specific rhythmic characteristics, is not clear. To address these questions, we considered a more general form of interpersonal coordination, implemented during joint artmaking. This is a non-typical context for interpersonal coordination, not required for task success, and smoother and more loosely-structured than more standard forms of synchronous coordination. Therefore, comparing interpersonal coordination with non-coordination during shared painting, could help reveal general social-emotional reactions to coordination. To gain a more ‘naïve’ perspective we focused on children, and staged coordinated and non-coordinated art interactions between an adult and a child, asking child observers to judge various variables reflecting the perceived bond between the painters. We found an overall stronger perceived bond for the coordination condition. These results demonstrate that even a non-typical form of interpersonal coordination could be attributed with positive social and emotional qualities, a capacity revealed already in childhood, with possible implications for development.

## Introduction

Many human activities are performed collaboratively. Such interactions often involve coordinated movements between interacting individuals. A special and well-studied case of interpersonal coordination is synchrony, whereby the coordinated movements are very strictly matched in time. This occurs during various joint activities ranging from simply walking together^1^ or engaging in a conversation^2^ to rowing as a team^3^ or playing in an orchestra^4^. In many cases, interpersonal synchronization is a basic requisite for the successful accomplishment of the joint activity. For example, for players in the same string section within an orchestra to produce coherent music, the individual bow movements must be continuously aligned in space and time.

Beyond such a practical role of synchronous coordination in enabling the basic execution of joint action, it has been discovered that the very experience of interpersonal synchrony appears to positively impact interacting individuals both socially and emotionally, further enhancing the quality of the interaction and even subsequent interactions^5–8^. These effects include enhanced feelings of belonging^9^, mutual understanding and fondness^10^, empathy^11^, and deeper comprehension of the emotional intentions of the synchronized collaborator^12^. Synchrony experience may also promote trust^13,14^, influence the memory of the interpersonal interaction^15^, and improve subsequent cooperation between interacting adults^15^ as well as children^16^.

Interestingly, even external adult observers witnessing other individuals engaging in synchronous interpersonal interaction, readily attribute to these individuals increased rapport^10,17^, entitativity^17,18^, affiliation^19^ and empathy^20^. Similar effects have also been shown in infants, who deemed asynchronous partners to be nonaffiliates^21^ or expected imitators to approach and affiliate with those who they imitated^22^, and in toddlers, who were able to make inferences about others’ affiliation based on movement synchrony already at 15 months of age^23^. These perceived effects of synchrony reveal how deeply ingrained the notion of interpersonal synchrony is, and its associations with positive social and emotional interaction. Thus, external impressions of observed interpersonal synchronous interactions can provide a useful readout of the coordinated experience.

The focus on the social and emotional impact of interpersonal synchronous coordination has mostly addressed discrete and punctuated forms of interaction, as often occurs in real life. This common type of coordination is characterized by uniformly interspersed distinct units of movement, such as tapping^9^, rocking^24^, and swinging^16,25^. In contrast, much less is known about smooth, irregular forms of interpersonal coordination, and their impact on interacting individuals. Such smooth and non-rhythmic movements may occur, for example, during artistic activities, such as painting. Although painting is typically an individualistic activity, it can be performed jointly, for instance, during group art lessons or in an art therapy context^26^. Interpersonal coordination during joint painting presents two unique features. First, it offers an opportunity to explore less structured forms of interaction, and to thus generalize the impact of synchrony experience to more general aspects of coordination. Second, the nature of the activity itself is improvisatory, has no defined purpose, and is open-ended, unlike, for example, rowing or rocking, or playing a notated piece of music together. As such the intentions of the interacting individuals are fluid and the entire interaction is dynamic and open to interpretation. The combination of both the smoothness and improvisatory nature of the interaction provides a unique context for the embodiment of interpersonal coordination. Thus, the social-emotional effects linked to synchronous interpersonal coordination can be dissociated from its functional and defined role in enabling the basic performance of the joint activity.

In another study to be published elsewhere, we have found that joint painting activity by two adults is perceived by external observers to be of higher quality when the painting movements are coordinated between the two painters compared to non-coordinated movements. This finding suggests that the perception of interpersonal coordination is much more general than previously thought, encompassing discrete and smooth forms of synchrony. Interestingly, a similar judgement of quality was made also by children observing the interaction, suggesting that the broad positive connotations of coordinated interaction are established already in early age. To better understand how interpersonal coordination is perceived by children, we sought in the current study to examine detailed aspects of how children judge five different aspects that could emerge from joint artmaking: empathy, social connection, closeness, similarity, and quality of interaction.

Previous work has demonstrated a capacity for synchronous interpersonal coordination experience to enhance each one of these social and emotional aspects of interaction. As described, synchronization has been shown to improve the understanding of other people’s emotional intentions^27^, as well as increase the ability to experience compassion and exhibit altruistic behavior^28^, which together may underlie enhanced *empathy*^12,29^. Synchronization can effectively increase the *social connection* among individuals sharing a synchronized experience^19^. Synchronization raises mutual understanding and fondness, enhancing perceived *closeness*^10,17,30^ and *similarity*^30^. We thus hypothesized that these effects should generalize to the smoother and subtler form of coordination introduced during joint painting, and be recognized by child observers. This, and our preceding study that is currently under consideration, are the first to explore the perception of this non-rhythmic, smooth and subtle form of interpersonal coordination in children.

## Materials and methods

### Participants

Participants were typically developing 9–11-year-old children, with an average age of 10.4 years (SD = 0.9). Parents were recruited to the study through ads that were posted on social media by the researchers (i.e., convenience sampling) and through parents of previous participants (i.e., snowball sampling). Altogether 20 boys (33%) and 41 girls (67%) participated by completing an online questionnaire that included closed-ended questions. Additional eight participants did not complete the questionnaire and accordingly, their responses were omitted from the study.

The study was initially conducted in Hebrew, but due to difficulties in recruiting a sufficient number of participants, it was translated into English, enabling the participation of additional participants from the USA and Australia. The Hebrew version of the questionnaire was completed by 53 participants from Israel (87%). The English version was completed by eight participants (13%), including four from Australia, two from the USA, and two from Israel who preferred to respond in English.

### Procedure

Parents of prospective participants were asked to access the Calendly (https://calendly.com) appointment scheduling software to choose a convenient date and time to conduct a telephone conversation with the researchers. At the appointed time, one of the researchers contacted the parents by telephone to provide an in-depth explanation of the study. The researcher double-checked the child’s age, and explained that the research will be conducted online and anonymously, and will consist of two short videos followed by a closed-ended questionnaire. The researchers provided also further instructions about how the questionnaire should be completed. At the end of the telephone conversation the researcher sent the parents a link to the online questionnaire via Qualtrics (https://www.qualtrics.com) online software. This program was used for gathering the research data.

Once the link was opened, the parents were first asked to give their informed written consent for their child’s participation in the study and to indicate the birth date and gender of their child. Next, the child was asked to work independently, without the assistance of family or friends. They were asked to view two short 90-s videos prepared by the researchers and to then fill in a questionnaire. As soon as the child finished filling in the questionnaire, the parents were asked to provide written feedback regarding the procedure of the study and whether there were any special events that occurred while the child was participating in the study, and submit the questionnaire.

### Research material

#### Videos

**Figure 1:**
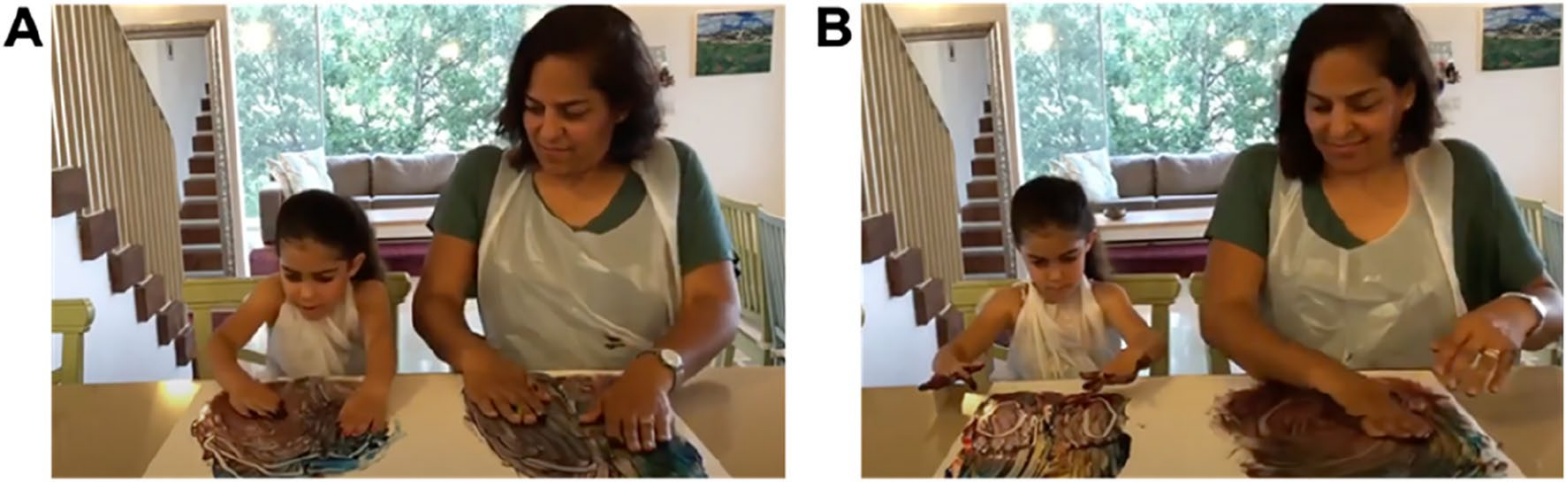
(**A**) A snapshot from the coordinated joint painting video. (**B**) A snapshot from the non-coordinated joint painting excerpt.

We prepared two videos of a woman and a 6-year old girl engaging in a shared hand painting process (Fig. 1; video excerpts are included in the Supplemental Material). Each video was shot at 30 frames per second, lasted 1 min and 40 s and showed one of two women (named Kara or Jackie in the English version, and Tal or Shani in the Hebrew version) interacting with one of two girls (named Tara or Becky in the English version, and Gal or Adi in the Hebrew version). In the coordination procedure, the woman adjusted her hand movements to match those of her young drawing partner. In the non-coordination procedure, the woman did not do so. A counterbalance was introduced between the woman-girl pairs so that half of the participants viewed Kara and Tara perform the coordination procedure, and Jackie and Becky perform the non-coordination procedure, and for the other half the pairs were swapped with regard to coordination/non-coordination. The two videos were presented to the participants in a random order.

#### Movement analysis of the interaction

To analyze movements during joint painting, video data were extracted and filtered by a video-based 2-D motion analysis system Kinovea® 0.8.27. Semi-automatic object tracking allowed us to measure distance and velocity, and to export data files for further analysis^31,32^. We manually selected a region of interest (ROI) around the middle finger of the right hand of each performer, and a 2D movement was then semi-automatically tracked. We applied grid-based calibration as a coordinate system, enabling measurements in the plane of motion not aligned with the camera.

**Figure 2:**
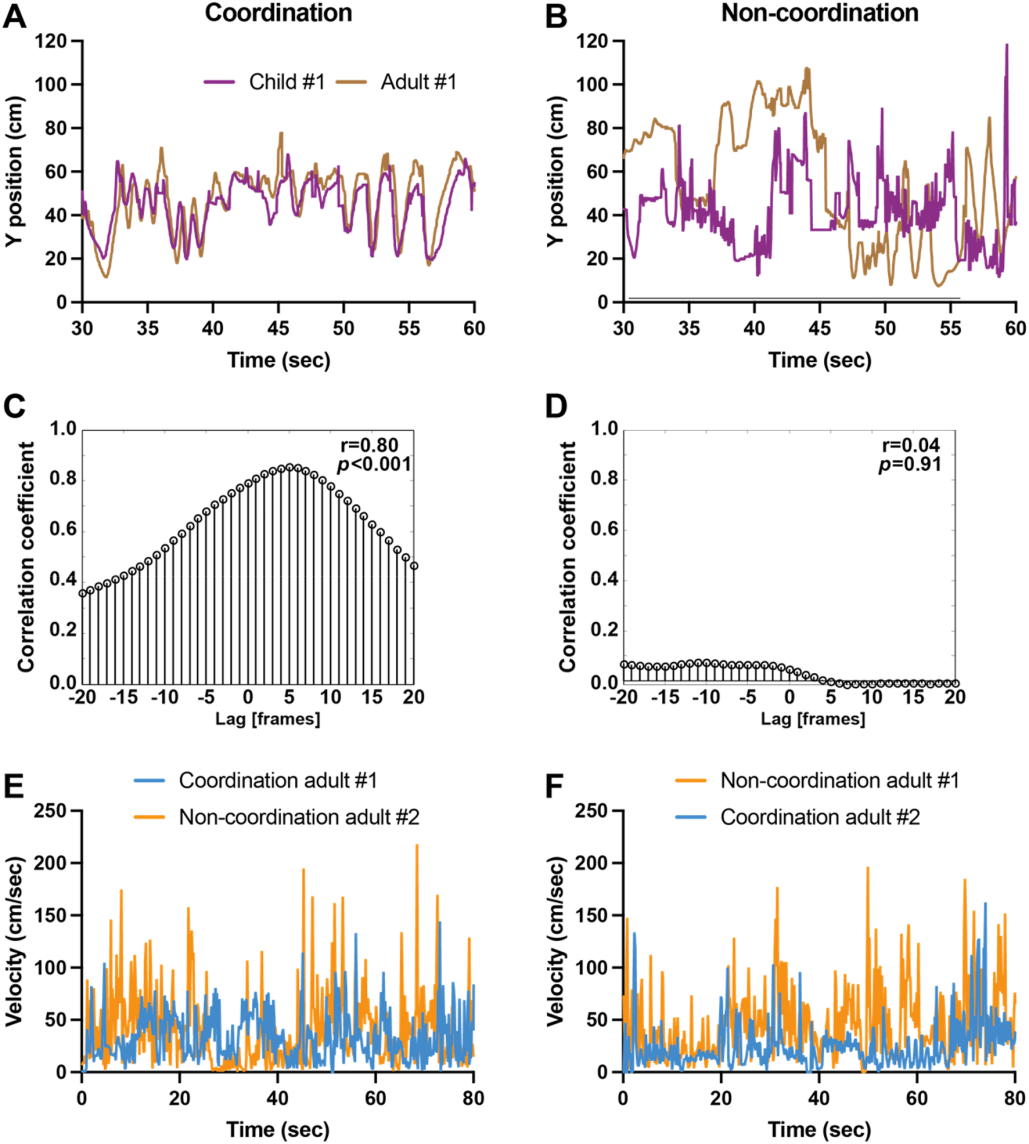
(**A**) Coordinated Right-hand movement on the y-axis for coordinated condition throughout video recording for girl and woman. The peak occurs after a 5 s lag. the girl is guiding the movement (**B**). Non-coordinated Right-hand movement on the y-axis for the non-coordinated condition throughout the video recording for girl and woman. (**C**) Cross-correlation between movement on the y-axis for coordinated condition. (**D**) Cross-correlation between movement on the y-axis for non-coordinated condition. (**E**) Right-hand velocity of the two adults, one during coordinated painting and the other during non-coordinated painting. (**F**) Right-hand velocity of the two adults, one during non-coordinated painting and the other during coordinated painting.

We calculated the hand position in the x and y-axes (centimeters) versus time (seconds). We plotted the hand position in the y-axis (centimeters) versus time (Fig. 2A, B). Then we conducted linear cross-correlation analysis, which provides a measure of overall coordination between participants, using the Matlab xcorr function (Mathworks, Natick, MA, USA), at lags k = 0, ±1, ±2,…, ±20 s (see^33,34^). Values close to 0 indicate that the position components were uncorrelated, while values close to 1 indicate that the position components were correlated (see Fig. 2C, D). In order to reduce jitter (due to imprecise tracking, for example), we replaced each 5 frames (166 ms in duration) with a single position value, which was the centroid of hand positions over these 5 frames. Then we calculated adult hand velocity in all four videos by dividing the Euclidean distance between each two consecutive centroids by 166 ms, and plotted velocity for adult #1 during the coordination condition in comparison to adult #2 in the non-coordination condition (Fig. 2E), and for adult #2 in the coordination condition vs. adult #1 in the non-coordination condition (Fig. 2F). For the first set (Fig. 2E) we found no significant difference in velocity between the two conditions (Mann Whitney *p* = 0.2955). However, for the second set (Fig. 2F) the differences were significant (Mann Whitney *p* < 0.0001) with a much higher median velocity for non-coordination (M = 38.35) compared to coordination (M = 19.69).

#### Questionnaire

After watching the two videos, the participants were asked to complete a 15-item questionnaire. These were divided into 5 sections relating to the five dependent variables examined in the study: empathy, social connection, closeness, similarity, and quality of interaction. The language and format of each item were adjusted to suit children. The closed-ended answers were presented in writing and through illustrations. All questionnaire items were adapted from various previously validated studies, as detailed below. The English version of the questionnaire is included in the Supplemental Material.

##### Empathy

Four questions were taken from the 17-item Empathic Resonance Scale questionnaire^35^ and adapted to children. For example, “Which woman had a better understanding of what the young girl felt and thought?” The answers included gradual comparative scales with four options, such as: (a) Kara did, much more than Jackie; (b) Kara did, slightly more than Jackie; (c) Jackie did, much more than Kara; and (d) Jackie did, slightly more than Kara. In each question, preference for the researcher in the coordinated painting video was rated 1, while preference for the researcher in the non-coordinated painting video was rated 0. A one-sided binomial test was then conducted for a 50% proportion. In addition, an overall perceived empathy score was calculated by averaging the individual question scores for each participant. The hypothesis was examined through a one-sided Wilcoxon signed ranks test that compared the median to 0.5.

##### Social connection

A single item was presented based on the Harris and Corriveau^36^ model for examining young children’s social connection through label imitation. Here, we used verbal imitation as a way of tallying children’s social connection towards the two women protagonists. Participants were shown pictures of the two women protagonists from the videos, each linked to a quote attributed to that woman about the shared activity. The participants were asked to choose which of the two sentences is correct in their opinion. The two sentences, however, had the same meaning: “Painting is a fun activity” and “Painting is an enjoyable activity”. We piloted this sentence and its specific wording prior to running the study to make sure that children did not have a preference towards one sentence over the other, and to make sure that they clearly perceive the two sentences as having the same meaning. According to the model, when children are confronted with a conflict in choice, they will choose the statement presented by the person whom they would feel the most connected to. A preference for the woman who appeared in the coordination video was rated 1, while a preference for the other woman was rated 0. A one-sided binomial test was conducted for a 50% proportion.

##### Closeness

Two questions were presented based on the Inclusion of Other in the Self (IOS) Scale by Aron et al.^37^, and our previous implementation of this scale^30^. The item included six pairs of circles. For each pair, the two circles were positioned at decreasing distances from one another (from far away to complete merging). The questionnaire was adapted to third person singular, and the circles contained the names of the woman and her young partner. Participants were asked to mark the pair of circles that most resembles the closeness between the adult and the child in the coordinated drawing, and then to mark the pair of circles that most resembles the closeness of the two in the non-coordinated drawing. These two questions were presented to participants in a random order. To calculate the difference in closeness between the two types of drawing, the coordination score was subtracted from the non-coordination score. The differences, which ranged from -5 to 5, were converted into scores between 0 and 1. For example, if the two pairs were given the same score for closeness, this would result in a difference of 0, to be transformed to 0.5 on the converted scale. A one-sided Wilcoxon signed ranks test was conducted to compare the median to 0.5.

##### Similarity

A self-reporting questionnaire for measuring similarity^30^ was employed. This questionnaire was designed to examine the degree of perceived similarity between children and their interacting partner, with particular emphasis on physical similarity and similarity in character and hobbies. The reliability of the original questionnaire measured by Cronbach's α was 0.73. Five out of the six questions in the original questionnaire were rephrased in the third-person singular. For example: *Which of the two pairs were more similar to each other?* The answers included gradual comparative scales with four options, such as: (a) Kara and Tara were much more similar than Jackie and Becky; (b) Kara and Tara were slightly more similar than Jackie and Becky; (c) Jackie and Becky were much more similar than Kara and Tara; and (d) Jackie and Becky were slightly more similar than Kara and Tara. For each question in this section, preference for the pair in the coordinated painting video was rated 1, whereas preference for the pair in the non-coordinated painting video was rated 0. A one-sided binomial test was conducted for a 50% proportion. As an overall score of perceived similarity, the average question score per participant was calculated. The hypothesis was examined through a one-sided Wilcoxon signed ranks test that compared the median to 0.5.

##### Quality of interaction

The AT-WAI Questionnaire^38^ originally developed for examining therapeutic alliance during art therapy, was employed. The reported reliability of the questionnaire (Cronbach's α) was 0.78. External validation was measured through the Pearson correlation coefficient for the bond component in the original WAI, and was r = 0.37 (*p* < 0.000). Out of this questionnaire, three questions from the *art experience* section were adapted to children and included in the present study. For example: *Which of the two girls enjoyed herself more while painting?* The participants were asked to choose from the following four options: (a) Tara enjoyed herself much more than Becky; (b) Tara enjoyed herself slightly more than Becky; (c) Becky enjoyed herself much more than Tara; and (d) Becky enjoyed herself slightly more than Tara. For each question in this section, preference for the girl in the coordinated painting video was rated 1, while preference for the girl in the non-coordinated painting video was rated 0. A one-sided binomial test was then conducted for a 50% proportion. An overall average ranging between 0 and 1 was calculated for the entire section for each participant. The hypothesis was examined through a one-sided Wilcoxon signed ranks test that compared the median to 0.5.

In addition to the analysis of individual questionnaire items and sections, an overall score was derived for each participant by averaging over all section scores. One-Sided Paired T-tests were conducted to compare this score’s average to 0.5.

#### Assessing the non-verbal behavior of the adult–child pair

In order to assess the non-verbal behavior of the adult–child pair we coded the time spent by child and adult looking towards their painting partner or towards their partner’s sheet during the joint painting. The data was annotated in ELAN which is an open-source and free multimodal annotation program developed by the Max Planck Institute for Psycholinguistics in Nijmegen^39,40^. Coding for looking time was performed when the adult or child was looking at their partner or her painting sheet, and is reported as a percentage of the total video duration. We did not code looking time towards other directions. The coding of looking times of adult to child and adult to child’s sheet, was performed in conjunction with the empathy measures, and child to adult and child to adult’s sheet, in conjunction with the quality of interaction measures. These were performed for both coordination and non-coordination video versions and then averaged and transformed into percentages. Coding was carried out independently by two experienced researchers. One of the coders coded the entire footage, and the second coded 50% of it. The inter-rater reliability was 0.99.

#### Addressing possible confounds

In order to examine whether participants may have had a distinct preference (i.e., bias) for one of the adults in the videos, or for one of the adult–child pairs, statistical analyses were conducted comparing the scores for each dependent variable between the two adult–child pairs, regardless of the coordination or non-coordination condition. Wilcoxon ranks sum tests for independent samples were conducted for the empathy, closeness, similarity, and quality of interaction measures, and a Chi-Square test for independent samples was performed for the social connection section.

To examine whether the age or gender of participants affected the results, the participants were divided according to age (above vs. below 10.5 years) or gender (boys vs. girls). Here too, Wilcoxon ranks sum tests for independent samples were conducted for the empathy, closeness, similarity, and quality of interaction measures, and a Chi-Square test for independent samples was performed for the social connection section.

In addition, to make sure that children are not responding based on demand characteristics (i.e., how they think they are expected to respond, rather than how they truly feel), we ran a separate control experiment, with a small sample of children (N = 15; average age 10.7 years, SD = 0.9; 4 boys and 11 girls) who did not participate in the main study. After watching the two coordinated and non-coordinated video excerpts, they were asked the following question regarding the two adults: “Who do you think is smarter?” The coordination condition is not expected to influence the children’s choice of who is smarter. However, if a bias is found in the children’s responses, then this could indicate a role for demand characteristics.

### Ethics

Following the introductory telephone conversation between the parent and the researcher, the parent was asked to sign a written informed consent form via Qualtrics. Moreover, the researcher emphasized that the child is not obligated to participate in the study and may stop their participation at any stage. Anonymity was ensured by separating the list with identifiers from the data files and keeping the results in a locked, password protected file. This study, which is part of a larger research project, was approved by the Ethics Committee at the University of Haifa (approval #354/20). We confirm that all research was performed in accordance with relevant guidelines/regulations, and that informed consent was obtained from all participants and/or their legal guardians. Informed consent for publication of identifying information/images in an online open-access publication was granted from the adult and child experimenters.

## Results

**Figure 3:**
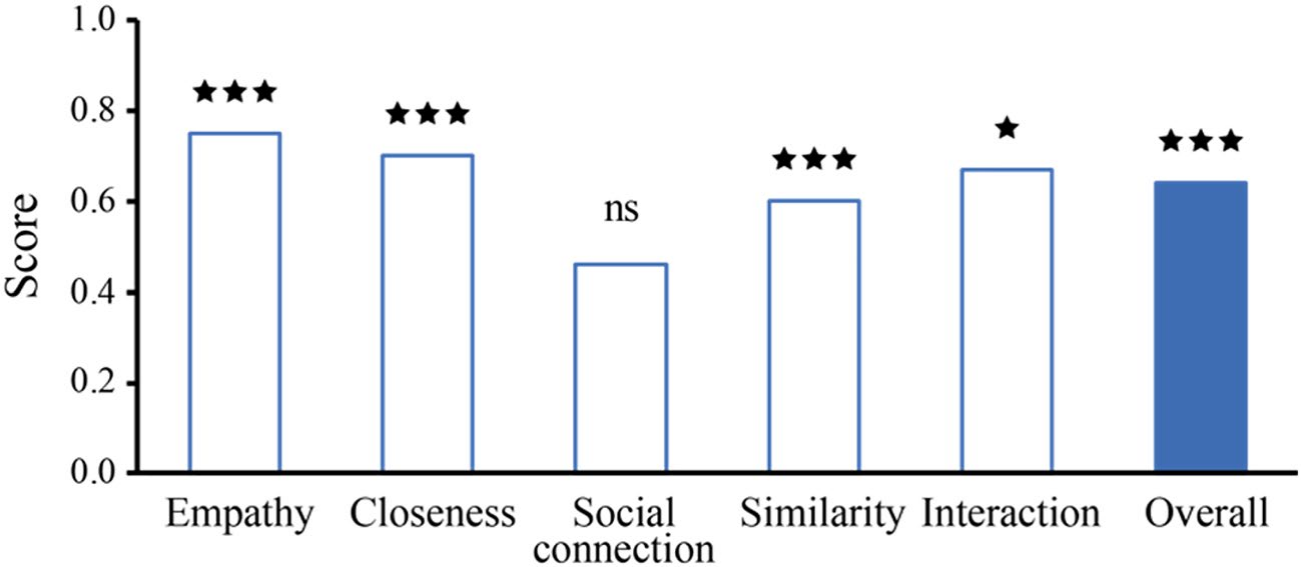
Summary of results.

The responses of child participants to videos showing a woman and girl painting together in coordination vs. non-coordination (Fig. 1) were examined along the five main dimensions of the study: empathy, social connection, closeness, similarity, and quality of interaction. Participants perceived the two interactions differently with respect to four out of the five dependent variables measured (Fig. 3).

### Empathy

As hypothesized, participants perceived the adult engaged in coordinated painting as more empathetic than the adult performing non-coordinated painting. This was indicated by an overall averaged empathy score with an observed median of 0.75 (*p* < 0.001), and a significantly higher than 0.5 rating of each individual question (Table 1).

**Table 1:** Empathy section results.

Question	Observed proportion*	Test statistic (Z)	1-Sided significance
Who better understood the child’s painting?	.89	6.02	< .001
Who better understood what the child felt and thought?	.67	2.69	.004
Who was more attentive to the child?	.90	6.27	< .001
Who was more interested in the child’s painting?	.77	4.48	< .001

*Observed proportions > 0.5 indicate a preference for the coordinated painting adult.

The adults spent a very low fraction of their time looking *towards the child* in both the coordination (M = 2.4%, SD = 1.03%) and non-coordination (M = 0%, SD = 0%) conditions. However, we found, in retrospect, that in the coordination condition the adults spent substantially more time looking towards *the child’s sheet* (M = 97.1%, SD = 0.4%) compared to the non-coordination condition (M = 2.3%, SD = 2.03%).

### Social connection

The hypothesis whereby the participants would perceive the adult in the coordinated art activity as more socially connected to them was not supported (observed proportion = 0.46, *p* = 0.739).

### Closeness

As hypothesized, participants perceived the adult and child in the coordinated interaction as being *closer* to one another with an observed median of 0.70 (*p* < 0.001).

### Similarity

As hypothesized, the adult and child in the coordinated art activity were perceived as being more *similar* to another compared to the pair in the non-coordinated activity, as indicated by the averaged similarity score (Observed median = 0.60, *p* < 0.001). Specifically, four out of the five questions that composed this overall similarity measure showed a significantly higher than 0.5 rating (Table 2).

**Table 2:** Similarity section results.

Question	Observed proportion*	Test statistic (Z)	1-Sided significance
Which pair generally looks more similar?	.56	0.90	.185
Which pair has more common interests?	.67	2.69	.004
Which adult reminds you more of the child she paints with?	.64	2.18	.015
Which adult is more similar in character to the child she paints with?	.66	2.43	.008
Which adult likes similar paining styles to the ones the child she paints with?	.75	3.97	< .001

*Observed proportions > 0.5 indicate a preference for the coordinated painting adult.

### Quality of interaction

As hypothesized, the quality of the interpersonal interaction between the adult and child engaged in coordinated joint painting was perceived as better and more enjoyable compared to the non-coordinated condition, as indicated by the averaged observed median of 0.67 (*p* = 0.017). In particular, three out of the four questions in this section received scores that were significantly higher than 0.5 (Table 3).

**Table 3:** Quality of interaction section results.

Question	Observed proportion*	Test statistic (Z)	1-Sided significance
Which child was more comfortable with the painting activity?	.67	2.69	.004
Which child felt freer during the painting activity?	.49	−0.13	.551
Which child enjoyed the painting activity more?	.62	1.92	.027

*Observed proportions > 0.5 indicate a preference for the coordinated painting adult.

The child spent very little time looking *towards the adult* in both the coordination (M = 0%, SD = 0%) and the non-coordination (M = 0.64%, SD = 0.9%) conditions. Similarly, the child did not look a lot at *the adult’s sheet* in both the coordination condition (M = 1.09%, SD = 0.46%) and the non-coordination condition (M = 3.8%, SD = 1.67%).

In summary, four of the five specific hypotheses were supported by the results (Fig. 3). In addition, the five measures were combined into one general variable relating to the overall perceived emotional bond between the adult and child in the painting activities. At this general level as well, the coordinated activity was perceived as reflecting a better emotional bond between the adult and child compared to the non-coordinated one (observed mean = 0.64, *p* < 0.001; Fig. 3).

To address the possibility of confounds due to an inherent preference for one of the two adults or adult–child pairs in the videos, participant responses were reordered according to the protagonists in the videos, ignoring whether they had engaged in a coordinated or non-coordinated joint painting interaction. No preference was found for a particular adult–child pair (Table 4).

**Table 4:** Data rearranged to compare between one adult–child pair and the other.

Section	Test statistic (Z)	2-Sided significance
Empathy	−0.07	.943
Closeness	−1.93	.053
Social connection*	2.32	.127
Similarity	0.86	.389
Interaction	−1.16	.246

*For the Social connection measure the test statistic was χ^2^ and not Z.

To probe a possible age or gender effect, responses were once more reordered, this time according to age (above vs. below 10.5 years) and then gender (boys vs. girls). Here too, no significant relationship was found between the participants’ age (Table 5) or gender (Table 6) and the choices made by the participants.

**Table 5:** Data rearranged to compare between responses of participants under vs. over 10.5 years of age.

	Under 10.5			Over 10.5			*p*
	*M*	*SD*	Median	*M*	*SD*	Median	
Empathy	0.78	0.22	0.75	0.84	0.22	1.00	.219
Similarity	0.71	0.27	0.80	0.60	0.25	0.60	.061
Interaction	0.53	0.30	0.67	0.67	0.38	0.67	.406
Closeness	0.66	0.18	0.70	0.65	0.19	0.70	.889
Social connection	0.48	0.51	0.00	0.43	0.50	0.00	.692
Overall	0.63	0.14	0.63	0.64	0.20	0.65	.813

**Table 6:** Data rearranged to compare between boy and girl participant responses.

	Boys			Girls			*p*
	*M*	*SD*	Median	*M*	*SD*	Median	
Empathy	0.83	0.22	1.00	0.80	0.23	0.75	.745
Similarity	0.66	0.28	0.70	0.65	0.25	0.60	.881
Interaction	0.53	0.37	0.67	0.63	0.33	0.67	.736
Closeness	0.67	0.19	0.70	0.65	0.18	0.70	.736
Social connection	0.55	0.51	1.00	0.41	0.50	0.00	.319
Overall	0.65	0.20	0.64	0.63	0.16	0.63	.501

In an additional smaller experiment designed to address a possible role for demand characteristics, we found that 9 (60%) of child participants considered the adult from the coordinated condition to be smarter, and 6 chose the adult from the non-coordinated condition as the smarter adult. We performed a Chi-square goodness-of-fit test to check whether these results differ significantly from an unbiased 50%/50% ratio (in our case, 46.67%/53.3%, since we had an odd number of participants). We found no significant difference between the expected and observed ratios (χ^2^(1) = 0.27, *p* = 0.62), suggesting that children did not follow coordination as a demand characteristic in their responses.

Finally, since we detected differences in adult velocity between the movements of adult #2 when painting in coordination and adult #1 when painting in non-coordination (see "Methods" section), we compared the responses of child participants who had viewed adult #1 in the coordination condition and adult #2 in the non-coordination condition (in this case the velocities were similar) and child participants who had viewed adult #1 in the non-coordination condition and adult #2 in the coordination condition (dissimilar velocities). We found that for all measures, the response distributions and median values were statistically indistinguishable between the two groups. Therefore, differences in coordination and non-coordination velocity that were unintentionally presented to a subgroup of the participants did not seem to have any impact on the results.

## Discussion

We have found that children observing an adult and child dyad painting together in a coordinated manner tend to perceive the painters as more empathic, more similar and closer to one another, compared to when they paint without particular coordination. In general, the children tended to judge the quality of the coordinated interaction to be better than the non-coordinated interaction. These results demonstrate the broad impact attributed to interpersonal coordination, even in the context of visual arts, which is not regularly associated with coordination between individuals. In particular, the enhanced social and emotional bonding perceived to exist between the interacting individuals, was evident despite the smooth and irregular form of coordination employed, which substantially differs from the standard discrete and rhythmic interaction considered in most previous work on synchronous interpersonal coordination both in adults^5–10^ and in children^16,21–23,30,41,42^. Our findings demonstrate that such a predisposition to identify even loosely-structured coordination within an interaction, and to judge it as indicative of social-emotional bonding, exists already in children and as such may play an important role during development.

Unlike music, which is rhythmically organized along the time dimension, and is often performed by a group, requiring synchronization between players, visual art, such as painting, is usually static, extending over space rather than time, and is typically created by a single individual. Nevertheless, a painting may still arouse in the observer physical and emotional sensations of the gestures and movements of the artist who had created it, even without actually seeing that artist in action^43^. Indeed, the notion of the observer blending in with the work of art, and internally imitating the motions and emotions embedded within it, was the initial inspiration for the concept of empathy, originally characterized by Lipps in the nineteenth century using the German term *Einfühlung*^44^. In the current study we took a step back and focused not on the finished piece of art but on the process of creating it, directly considering the painters’ actions. The joint painting provided a unique context of a coordinated interaction that is not typically associated with synchrony and does not require synchrony for its successful accomplishment. These features, made it possible to dissociate the net effects of coordination from other factors such as the timing-dependent or independent nature of the activity and the necessary conditions for its performance, and to generalize the notion of interpersonal coordination to the visual arts, thus opening new avenues for considering art-based interpersonal interactions. It would be interesting, in future work, to take a further step back and explore whether the mere movements of the interaction, even without the use of art materials may be sufficient for creating similar perceptual effects.

Interpersonal coordination in the visual arts may have specific implications for visual art therapy. Previous work has demonstrated how coordination in the form of synchronization may impact various other forms of therapy, including psychotherapy, music therapy, and drama therapy, enhancing the quality of the therapeutic bond^12,45^. In analogy, during art therapy sessions, shared client-therapist artwork is sometimes applied as a means of familiarization and evaluation^26,46^. One of the most familiar tools is Winnicott’s Squiggle Game, aimed at creating an initial “interview” with child clients during therapy^47^. Joint drawing and painting are also used to enhance the therapeutic bond and improve the therapist’s understanding of the client^48,49^. Our results suggest that enhancing coordination during shared squiggling or painting may strengthen the foundations of the therapeutic alliance. This direction should be further explored.

According to our findings, study participants perceived the adult in the coordinated painting condition as having a better understanding of the young girl’s painting and of her thoughts and feelings. The adult was also perceived as being more attentive towards the young girl and more interested in her painting. It is likely that watching the pair work in coordination led the participants to feel that a state of empathy, understanding, and mutual language exists between the two, whereby one’s hand movements are familiar, understood, and learned by the other, through means of observation and attention. Thus, coordination could be interpreted by the observers either as the outcome of preexisting empathy or as its inducer. Another possibility to consider is the enhanced looking time of the adult towards the child’s sheet, but not towards the child herself, in the coordination condition, which we found to unintentionally occur, perhaps as a means for achieving coordination by the adult. This could have potentially directed the participating children to judge the adult in the coordination condition as more attentive and interested in the child’s actions, compared to the non-coordination condition, and thus, more empathic. In future studies it will be important to dissociate between movements and looking during coordinated interactions, in order to determine their relative impact on how the interaction is perceived by observers.

A possible limitation of the study could stem from child responses being based on demand characteristics, in line with what they assume is expected from them (i.e., judging coordination as the desirable experimental condition and thus biasing their responses in favor of this condition). Although, no such bias was observed for the social connection variable or for the smartness variable in the additional follow-up study that we conducted, this remains a possibility.

The current research hypothesized that the participants would perceive the adult in the coordinated painting condition as more socially connected to them, yet the findings did not support this. The reason for this may stem from the method used for assessment. As described in the "Methods" section, participants had to choose between two dissimilarly phrased, but synonymous quotes attributed to the coordinating vs. non-coordinating adults (“Painting is a fun activity” and “Painting is an enjoyable activity”). Influenced by previous studies^36,50^, the rationale was that when asked to select which of the two quotes was more correct, the child observers would select the phrase attributed to the adult they felt better connected to. However, no preference for the synchronized adult was found, differing from previous studies that have shown synchronous interpersonal coordination to be linked to enhanced social connection^19^. It is likely that the difference in phrasing between the two adult quotes was too subtle, possibly creating confusion rather than bias among the participants. In addition, the question asked differed from the rest of the questions in the questionnaire, as it focused on a possible relationship between the adult character and the child participant rather than between the characters. It seems unlikely that enhanced social connection is associated only with synchronous coordination, and not with coordination, in general. Future studies could benefit from implementing a different type of measurement for examining this relationship, and clarifying the results.

With regards to *closeness*, the findings of this study indicate that the adult–child pair in the shared coordinated painting activity were perceived as being closer to one another compared to the pair in the non-coordinated activity. These findings are in line with previous studies whereby synchronous coordination between two individuals were shown to impact feelings of closeness between the pair^19,20^. Studies have also shown the reciprocal effect, whereby an increased feeling of closeness between random pairs is accompanied by an increase in interpersonal synchronized behavior^51^. In relation to the perceived closeness between people who are not directly connected, studies have shown that when participants observe synchronized people, they maintain certain beliefs about why these people are synchronized. One such belief is that people who move together do so because they share emotions or feelings of belonging and closeness^9,17^. Miles et al.^10^, for example, examined the degree of mutual understanding and fondness between pairs who walked side by side but with different degrees of synchronization, as perceived by the observing participants. The findings showed that greater synchronization in walking was associated with greater perceived closeness. Our current results suggest that perhaps underlying this effect is the increase in coordination, which may stem, in the case of synchrony, from a better rhythmic match. Future studies could therefore benefit from examining shared painting activities with varying degrees of coordination.

The fourth element examined in this study relates to *perceived similarity*. Our findings show that the child participants perceived the similarity between the pair in the coordinated activity as greater than between the pair in the non-coordinated one. This is consistent with previous studies, which found that children and adults who participated in a synchronized interpersonal interaction with random partners, perceived the similarity between themselves and their partners as greater than those who participated in an asynchronous activity^30,52,53^. Such results were found also in a study, in which the participants observed (but did not participate in) the interactions of pairs who moved in synchronization and pairs who did not^17^.

Meltzoff^54^ found that infants tend to imitate adult behavior, which leads them to feel similar to the imitated adult. This feeling among infants extends beyond the physical similarity of their behavior or movements to also include similarities in their interpretation of the other person’s psychological situations, such as perceptions and emotions. Meltzoff coined this type of representation as “Like Me” and viewed this as the starting point of social cognition. Similarly in the current study, the participants perceived the adult and child in the shared coordinated procedure as more similar to one another in their personalities, hobbies, and types of preferred paintings. This perceived similarity may stem from the perception extending beyond the similarity of movements to also include personal similarities. In other words, if the adult–child pair painted in a coordinated manner, then perhaps they were perceived as having additional similar traits as well as a similar personality and similar hobbies.

Finally, the current study found that the quality of the *interpersonal interaction* between the adult and child in the coordinated painting activity as better and more fun and the overall score of social and emotional bonding showed a clear preference for the coordination condition. Examining the child’s looking time towards the adult and towards the adult’s sheet in order to elucidate whether the participating children may have been influenced by the children’s looking patterns when they answer questions regarding the children enjoying themselves, feeling free and being comfortable, showed similar looking patterns between the two conditions in both measures, strengthening the notion that the preference for the coordination condition in this case stems mainly from the form of painting itself (coordination vs. non-coordination).

In summary, this study supports the claim that shared coordinated painting is perceived by child participants to be associated with greater empathy, closeness, similarity, and quality of the interaction when compared to non-coordinated joint painting. The study is unique as it examines these components within the context of art, specifically in relation to shared visual art activities. Similar to creating intentional synchronized moments during music, drama, and movement, coordination between painters could also play a potential role in interpersonal activities based on visual art. Moreover, although the current study focused on how shared coordinated painting activities are perceived by observers, not by the activity of the participants themselves, the findings have important implications; they emphasize how readily interpersonal coordination can be discerned, and show the extent of its influence even at the perceptual level, of merely observing an interaction without being part of it. In particular, the study shows that even relatively young children perceive coordinated and non-coordinated activities differently.

## Supplementary Information

Supplementary Video 1.

Supplementary Information 1.

Supplementary Information 3.

Supplementary Video 2.

## Data Availability

Data generated during this study are available and has been submitted as part of the Supplemental Material.
