# Supplementary material for: Children’s perception of interpersonal coordination during joint painting
Source: Sci Rep. 2022 Nov 7;12:18897. doi: 10.1038/s41598-022-22516-2 (PMC9640642; doi:10.1038/s41598-022-22516-2)
Supplement: Supplementary file 3 — Supplementary Information 3. [file 41598_2022_22516_MOESM3_ESM.pdf]

1. Parent information and consent form

הפקולטה למדעי הרווחה והבריאות

Faculty of Social Welfare & Health Sciences

الكلية لعلوم الرفاه والصحة

Questionnaire – co-painting

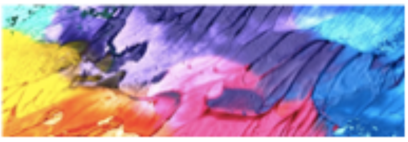

We invite typically developing children aged 9-11 and their parent to participate in a research study that looks at the interaction between a child and an adult during co-painting. This research is being conducted at the School of Creative Arts Therapies and the Department of Psychology at the University of Haifa.
Participation includes watching two short video excerpts portraying an adult and a child painting together, followed by an anonymous questionnaire regarding the interaction between them. In the first stage, the parent is asked to watch the videos and answer the questions, and in the second stage, the child is asked to watch the videos and answer the questions.
The estimated total time for each person to complete the study is about 10 minutes. Filling out the questionnaire can be done independently by the child using a computer and if there is a technical difficulty, the parent can assist.
Importantly, the child must work independently, without the parent's involvement.
We ask that you complete the study using a computer, in order to ensure an optimal view of the video excerpts and questions.
The study is approved by the Ethics Committee of the University of Haifa.

Thank you for your cooperation.

Dr. Tal-Chen Rabinowitch, The School of Creative Arts Therapies, University of Haifa
Rotem Abraham, The School of Creative Arts Therapies, University of Haifa
Naama Rachel Nissel Miller, The Department of Psychology, University of Haifa
Phone inquiries: 972-54-2567502, 972-52-4677565

הפקולטה למדעי הרווחה והבריאות

אוניברסיטת חיפה
University of Haifa
جامعة حيفا

Faculty of Social Welfare & Health Sciences

الكلية لعلوم الرفاه والصحة

Confirmation of my participation and the participation of my child in research on the subject: Dyadic communication in co-painting

I confirm that I have read the information presented to me and that the purpose of the research has been explained to me.
I understand that participation is by choice only, and if I refuse it will not have any consequences for me or my child in the future.
I understand that I/my child can cease participation at any stage, and this will not have any consequences for me or my child in the future.
I agree to my participation and the participation of my child in this study and I am prepared for the questionnaire to be used for research purposes only.

Please mark according to your choice:

O I **confirm** my participation and the participation of my child in the study

O I **do not** confirm my/ my child participation in the study

2. Parent questions

This part should be filled out by the parent

Personal details of my child

Month and year of birth of my child

Participation in the questionnaire will be conducted by

Month	Year	
⌄	⌄	Please choose:

- O My son
- O My daughter

3. Video presentation for child

This part should be filled out by the child

You are about to watch two short, 1.5 minute, video excerpts, followed by questions that relate to the excerpts.
We ask for your opinion only. There are no right or wrong answers.

We would like to remind you that the filling out this section is done by the child only, without the presence and participation of the parent.

We asked Kara and Tara, and also Jackie and Becky, to paint together.
Please watch the video excerpts, and answer the following questions.

Kara and Tara are painting together.
Kara is the adult and Tara is the child.

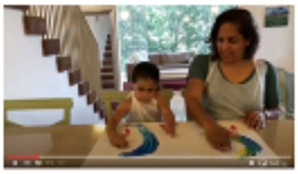

Jackie and Becky are painting together.
Jackie is the adult and Becky the child.

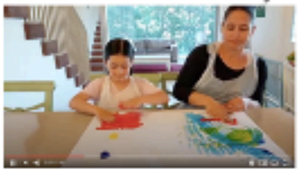

please confirm:

O I watched both video excerpts

4. Child questionnaire

Empathy

Please remember: There is no right or wrong answer, we only want your own personal opinion.

Who better understood the child's painting?

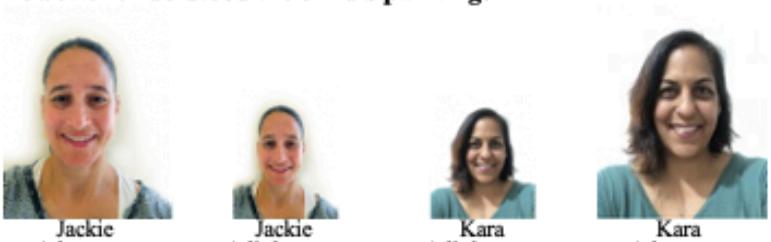

Jackie	Jackie	Kara	Kara
A lot more	A little more	A little more	A lot more
O	O	O	O

Who better understood what the child felt and thought?

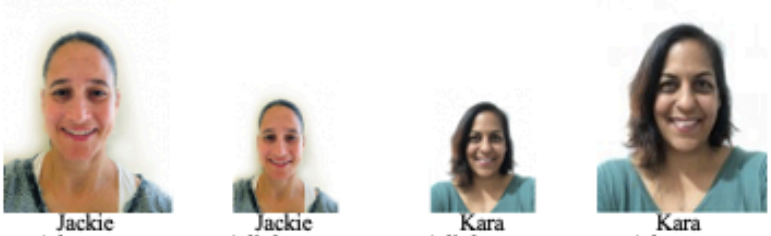

Jackie	Jackie	Kara	Kara
A lot more	A little more	A little more	A lot more
O	O	O	O

Who was more attentive to the child?

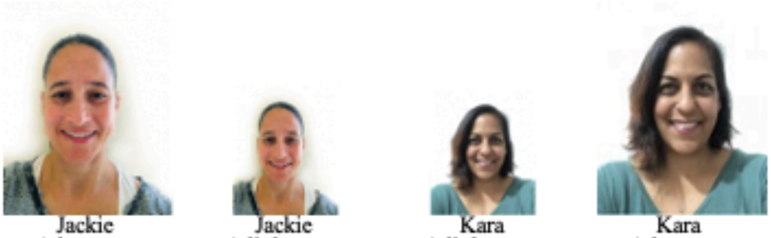

Jackie	Jackie	Kara	Kara
A lot more	A little more	A little more	A lot more
O	O	O	O

Who was more interested in the child's painting?

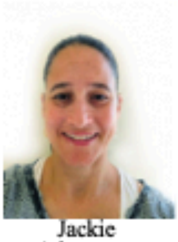
Jackie
A lot more
O

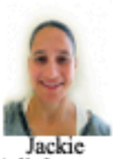
Jackie
A little more
O

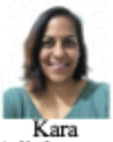
Kara
A little more
O

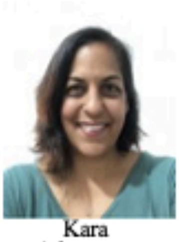
Kara
A lot more
O

Trust

Who do you think is right?

Please remember: There is no right or wrong answer, we only want your own personal opinion.

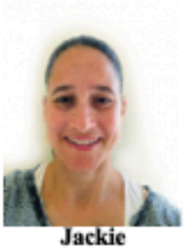

Jackie

Painting is
an enjoyable activity
O

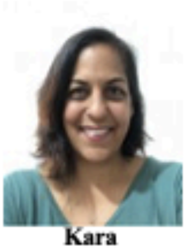

Kara

Painting is
a fun activity
O

Closeness

Out of the six pairs of circles, which best portrays how close Kara and Tara feel towards each other!
Please remember: There is no right or wrong answer, we only want your own personal opinion.

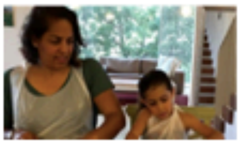

O 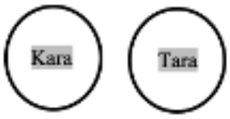

O 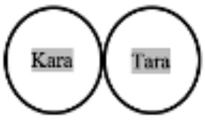

O 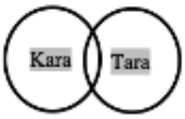

O 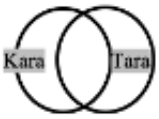

O 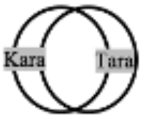

O 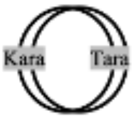

Out of the six pairs of circles, which best portrays how close Jackie and Becky feel towards each other?
Please remember: There is no right or wrong answer, we only want your own personal opinion.

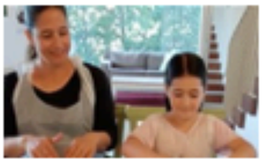

○ 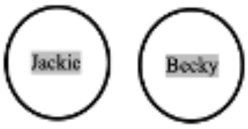

○ 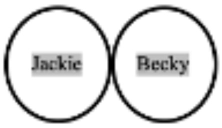

○ 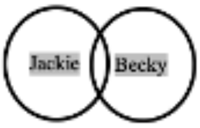

○ 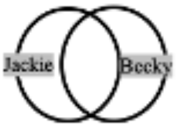

○ 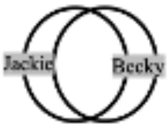

○ 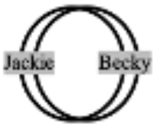

Similarity

Please remember: There is no right or wrong answer, we only want your own personal opinion.

Which pair generally looks more similar?

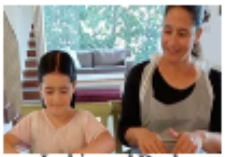

Jackie and Becky
A lot more
O

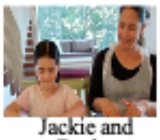

Jackie and Becky
A little more
O

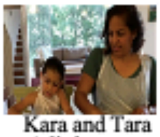

Kara and Tara
A little more
O

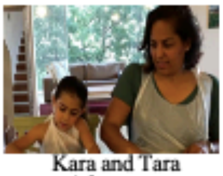

Kara and Tara
A lot more
O

Which pair has more common interests?

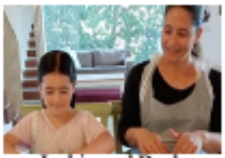

Jackie and Becky
A lot more
O

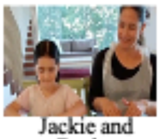

Jackie and Becky
A little more
O

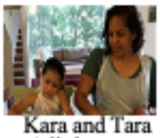

Kara and Tara
A little more
O

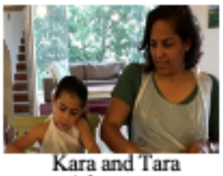

Kara and Tara
A lot more
O

Which adult reminds you more of the child she paints with?

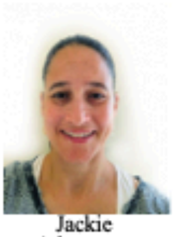

Jackie
A lot more
O

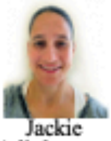

Jackie
A little more
O

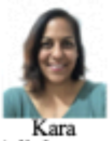

Kara
A little more
O

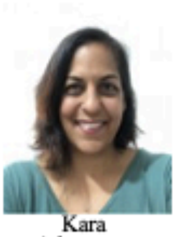

Kara
A lot more
O

Which adult is more similar in character to the child she paints with?

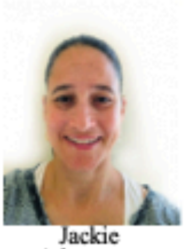
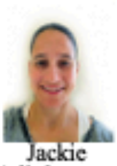
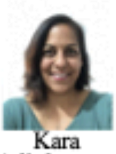
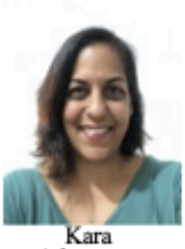

Jackie<br>A lot more	Jackie<br>A little more	Kara<br>A little more	Kara<br>A lot more
O	O	O	O

Which adult likes similar painting styles to the ones the child she paints with does?

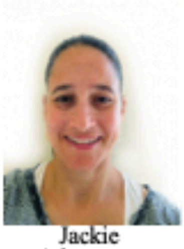
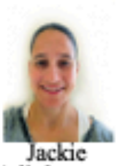
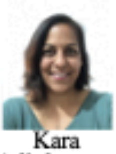
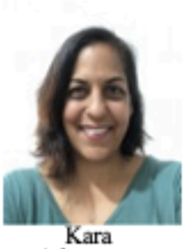

Jackie<br>A lot more	Jackie<br>A little more	Kara<br>A little more	Kara<br>A lot more
O	O	O	O

Quality of interaction

Please remember: There is no right or wrong answer, we only want your own personal opinion.

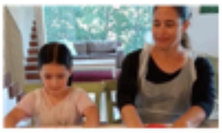
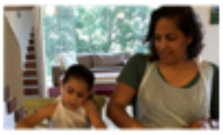

Which child was more comfortable with the painting activity?

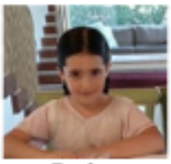
Becky
A lot more
O

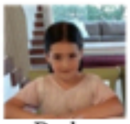
Becky
A little more
O

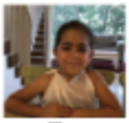
Tara
A little more
O

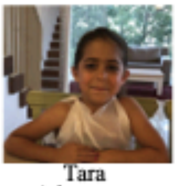
Tara
A lot more
O

Which child felt freer during the painting activity?

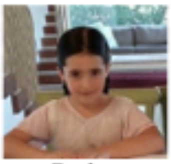
Becky
A lot more
O

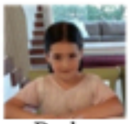
Becky
A little more
O

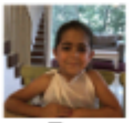
Tara
A little more
O

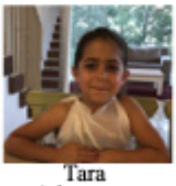
Tara
A lot more
O

Which child enjoyed the painting activity more?

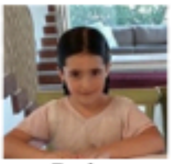
Becky
A lot more
O

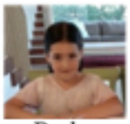
Becky
A little more
O

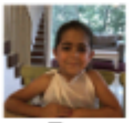
Tara
A little more
O

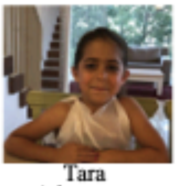
Tara
A lot more
O
